# A Multidisciplinary Basic Airway Skills Boot Camp for Novice Trainees

**DOI:** 10.7759/cureus.8766

**Published:** 2020-06-22

**Authors:** Lawrence Kashat, Bridgette Carter, Michael Archambault, Zhu Wang, Katherine Kavanagh

**Affiliations:** 1 Otolaryngology, University of Connecticut, Farmington, USA; 2 Otolaryngology, Connecticut Children’s Medical Center, Hartford, USA; 3 Anesthesiology, Connecticut Children’s Medical Center, Hartford, USA; 4 Population Health Sciences, University of Texas Health Science Center, San Antonio, USA; 5 Otolaryngology, Connecticut Children's Medical Center, Hartford, USA

**Keywords:** skills and simulation training, simulation in medical education, otolaryngology, anesthesiology, difficult airway management, resident education, simulation course, difficult airway, airway procedures, boot camp

## Abstract

Introduction: Otolaryngology and anesthesiology residents may be the first responders to airway emergencies, even in the first weeks of training. These residents may be unfamiliar with the armamentarium of airway maneuvers, the most basic of which may be lifesaving. Boot camp education has been demonstrated to be effective in multiple disciplines. In this study, we examine whether an immersive, multidisciplinary boot camp style simulation course leads to an improvement in novice airway provider confidence.

Methods: Novice otolaryngology and anesthesiology residents participated in an annual (2013-2018) multidisciplinary boot camp simulation course. Residents completed an anonymous pre- and post-test self-assessment tool reporting their confidence for airway skills and concepts from the curriculum. Responses were on a Likert scale from 1 to 5 (1=no familiarity, 5=extremely comfortable). We analyzed pre- and post-course participant self-reported comfort level with the airway management skills and concepts addressed in the course. Frequencies and percentages were reported. Fisher’s exact test was used to assess statistical significance at level 0.05.

Results: A total of 62 residents, including 50 anesthesiology residents and 12 otolaryngology residents, completed a post-test self-assessment tool. For all topics covered in the course, there was a statistically significant change in the percentage of residents who reported familiarity with the topic (p<0.001). This corresponded to an increase in self-reported comfort level and a decrease in non-familiarity or discomfort with the airway topics covered in the course. The greatest increase in percentage of residents reporting feeling comfortable or extremely comfortable with the task after completion of the simulation boot camp were all moderately advanced airway maneuvers (laryngeal mask airway [LMA] placement, flexible fiberoptic intubation, glidescope intubation, endotracheal intubation, and two-person mask ventilation). The greatest decrease in non-familiarity or discomfort was seen in moderately complex to advanced airway topics (fiberoptic intubation, glidescope intubation, intubating LMA, rigid bronchoscopy, cricothyroidotomy, tracheostomy, and laryngectomy).

Conclusions: Our data support the use of immersive surgical boot camp experiences to enhance resident familiarity and comfort and decrease unfamiliarity and discomfort with a wide variety of airway topics and maneuvers.

## Introduction

On their first day as an otolaryngology or anesthesiology resident, a trainee may be called upon as an airway “expert.” Concern regarding the uniform preparedness of surgical interns upon commencement of residency has been raised due to variability in medical school training and procedural preparedness [1]. Medical school training provides students with a broad knowledge base and with skills to succeed as general physicians, but standardized exposure to medical and surgical subspecialties is often limited. Students often participate in electives and sub-internships to gain further knowledge in special interests that they may have, but these experiences vary widely among trainees. The variability in exposure and experience with technically advanced or specialty-specific tasks is one reason many resident physicians may feel a great increase in anxiety as they transition from clerkship to direct patient care at the beginning of residency [2]. This is particularly relevant among surgical and procedural specialties because although students are required to pass standardized tests to successfully complete medical school, many, if not most, students have limited experience with the procedures that they will perform and eventually learn to master in residency.

Otolaryngology and anesthesiology residents are often the primary responders to airway emergencies. These experiences, especially among beginner and novice trainees, can be a source of great anxiety and stress. Novice otolaryngology and anesthesiology residents may be first responders to an airway emergency even within the first few weeks or residency training despite the fact that most of these early trainees may have limited to no experience with airway assessment and with performing airway maneuvers, the most basic of which may be lifesaving. In fact, a study of post-graduate year (PGY)-2 otolaryngology residents 87% reported airway management and needing to establish a surgical airway as a source of anxiety [3].

Traditional surgical education has revolved around an apprenticeship-based model in which novice trainees gain experiences under the supervision of senior residents and attendings. This model of training is reflected in the oft-cited mantra of “see one, do one, teach one,” a concept that may be supplemented and improved upon [3]. As part of an effort to bridge the experience gap that trainees may face at the start of residency, the American Board of Surgery and American College of Surgeons have suggested that all medical schools adopt a surgical pre-residency preparatory course to help medical students gain technical skills and improve comfort with procedures [4]. Within otolaryngology, most program directors have reported incorporating some simulation training as part of their residency curriculum and a wide range of simulations have been described encompassing a variety of clinical and surgical tasks [2,3,5].

The first month of the new academic year also poses significant safety issues for patients as there is an increase in mortality associated with new physicians taking on clinical responsibilities for the first time [6]. Simulation offers a safe, distraction-free and risk-free environment to immerse trainees in experiences they are likely to encounter during their training. In contrast to the knowledge gained from working with patients or performing procedures in the operating room, the safe environment afforded by simulation has the potential to improve the knowledge and comfort level of trainees with procedures without posing a risk to patients. In this study, we examine whether an airway simulation boot camp leads to an improvement in novice airway provider familiarity with the step-by-step approach to airway management described in the American Society of Anesthesiologists (ASA) difficult airway algorithm and with surgical airway techniques and modifications.

## Materials and methods

We developed a multidisciplinary boot camp simulation course for PGY-2 anesthesiology and otolaryngology residents to teach introductory airway knowledge and procedural skills. The course is performed over a full day and has been repeated annually since 2013. It combines short didactics with targeted simulation activities. Each simulation is facilitated by otolaryngology and anesthesiology faculty. The overriding concept driving the topics covered in the course is the ASA difficult airway algorithm and the course was designed so that participants learn each skill along the algorithm pathway. Adjunctive airway maneuvers are highlighted to demonstrate their importance in the management of both routine and challenging airways. Topics were included to address more complicated airway maneuvers and situations, including fiberoptic intubation, video laryngoscopy (glidescope intubation), intubating laryngeal mask airway (LMA), rigid bronchoscopy, cricothyroidotomy, tracheostomy, and laryngectomy. 

The data were prospectively collected and analyzed. Participants completed a pre- and post-course self-assessment tool to rate how comfortable they felt with airway tasks or conditions. They rated themselves along a Likert style continuum, which included “no familiarity”=1, “not comfortable”=2, “neutral”=3, “comfortable”=4, and “extremely comfortable”=5. We analyzed pre- and post-course participant self-reported comfort level with the airway management skills and/or concepts addressed in the course. Frequencies and percentages were reported, and Fisher’s exact test was used to assess statistical significance at level 0.05.

## Results

Between 2013 and 2018, a total of 62 incoming PGY-2 anesthesiology and otolaryngology residents participated in the airway simulation boot camp experience. Of these residents, 12 were otolaryngology residents and 50 were anesthesiology residents. Of the 62 participants, 60 completed both pre- and post-simulation self-assessment tool (97%) and two completed only the post-simulation self-assessment tool. As this was an anonymous self-assessment tool, there was no way to differentiate between the scores of the anesthesiology and otolaryngology residents.

**Figure 1:**
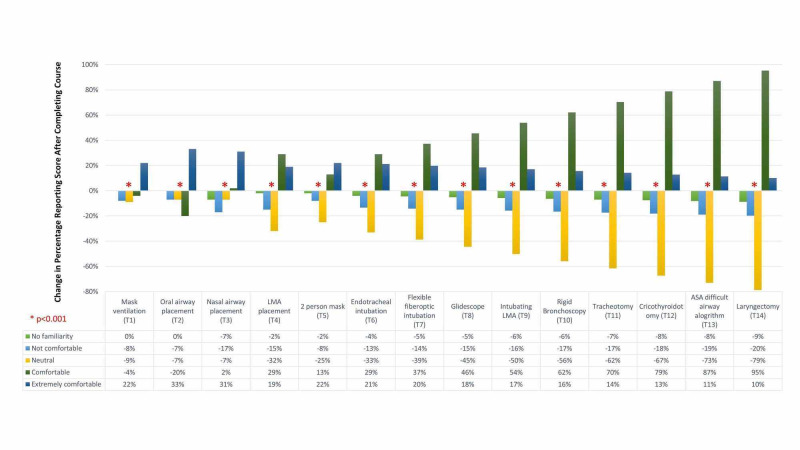
Change to percentage of novice otolaryngology and anesthesiology residents’ self-reported comfort and familiarity scores with simulated airway topics prior to and after completing an immersive simulation boot camp experience. For all topics covered in the course, there was a statistically significant change in the percentage of residents who reported familiarity with the topic (p<0.001). This corresponded to an increase in self-reported comfort level and a decrease in non-familiarity or discomfort with the airway topics covered in the course. Acronym key: LMA = laryngeal mask airway, ASA = American Society of Anesthesiologists.

**Figure 2:**
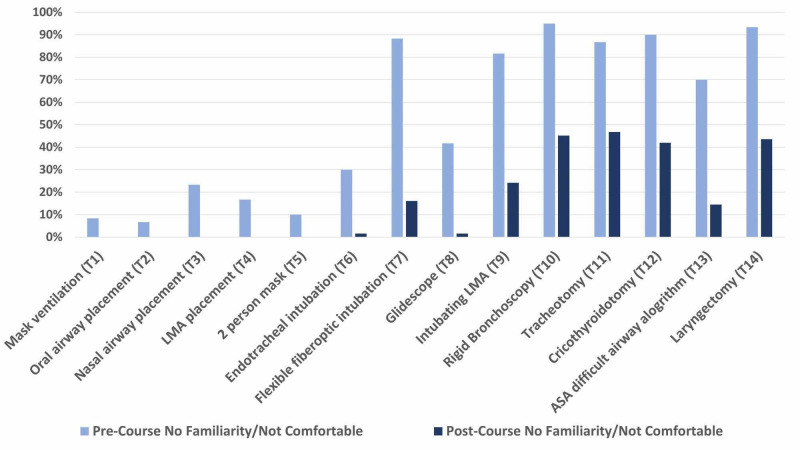
Percentage of novice otolaryngology and anesthesiology residents reporting no familiarity or feeling not comfortable with simulated airway topics prior to and after completing an immersive simulation boot camp experience. The five topics with the greatest percentage of residents reporting pre-simulation scores that reflected no familiarity or feeling not comfortable with the task were also the most complex: rigid bronchoscopy (95%), laryngectomy (93%), cricothyroidotomy (90%), flexible fiberoptic intubation (88%), and tracheostomy (87%)]. The five topics with the greatest decrease in percentage of residents who reported no familiarity or feeling not comfortable were all moderate to complex airway maneuvers and topics: flexible fiberoptic intubation (-72%), intubating LMA (-58%), laryngectomy (-49%), rigid bronchoscopy (-50%), and cricothyroidotomy (-48%). Acronym key: LMA = laryngeal mask airway, ASA = American Society of Anesthesiologists.

**Figure 3:**
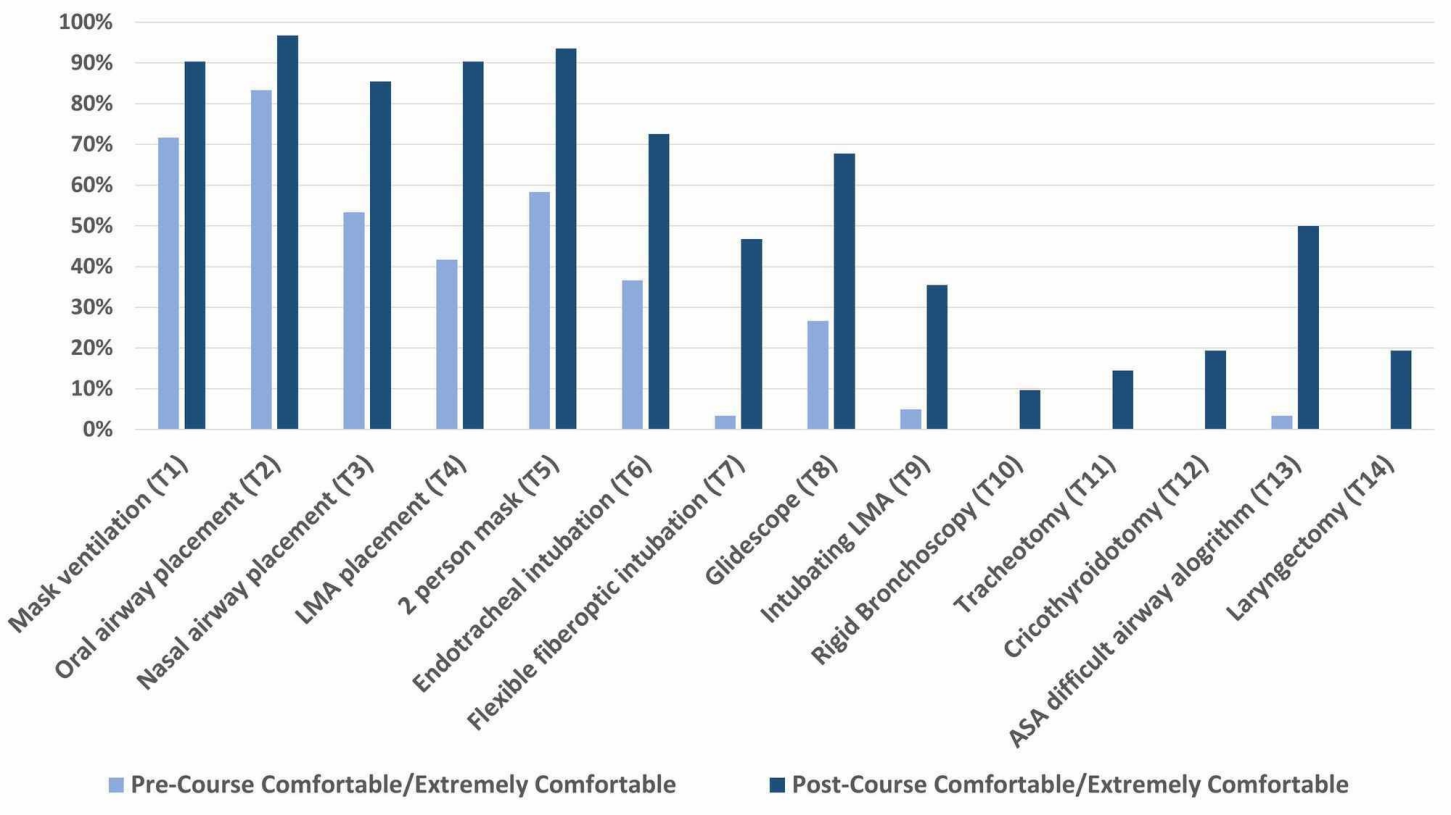
Percentage of novice otolaryngology and anesthesiology residents reporting feeling comfortable or extremely comfortable with simulated airway topics prior to and after completing an immersive simulation boot camp experience. The greatest increase in percentage of residents reporting feeling comfortable or extremely comfortable with the task after completion of the simulation boot camp were all moderately advanced airway maneuvers. This included LMA placement (+48%), flexible fiberoptic intubation (+44%), glidescope intubation (+41%), endotracheal intubation (+36%), and two-person mask ventilation (+36%). Acronym key: LMA = laryngeal mask airway, ASA = American Society of Anesthesiologists.

For all 14 tasks in the simulation boot camp, there was a change to the percentage of residents reporting familiarity with the airway topics covered in the boot camp course (p<0.001) (Figure 1). This corresponded to a decrease in the percentage of residents reporting no familiarity (1/5) or not comfortable (2/5) scores (Figures 1, 2) and an increase in the percentage of residents reporting comfortable (4/5) or extremely comfortable (5/5) scores (Figures 1, 3). 

The five topics with the greatest percentage of residents reporting pre-simulation scores that reflected no familiarity or feeling not comfortable with the task were also the most complex. These included rigid bronchoscopy (95%), laryngectomy (93%), cricothyroidotomy (90%), flexible fiberoptic intubation (88%), and tracheostomy (87%). Conversely, the topics with the fewest residents reporting feeling unfamiliar or not comfortable prior to participation in the boot camp were oral airway placement (7%), mask ventilation (8%), two-person mask ventilation (10%), LMA placement (17%), and nasal airway placement (23%).

The greatest increase in percentage of residents reporting feeling comfortable or extremely comfortable with the task after completion of the simulation boot camp were all moderately advanced airway maneuvers. This included LMA placement (+48%), flexible fiberoptic intubation (+44%), glidescope intubation (+41%), endotracheal intubation (+36%), and two-person mask ventilation (+36%) (p<0.001) (Figure 3). The mean increase in these topics was 41%. Interestingly, although LMA placement was one of the five topics that residents reported the lowest levels of pre-simulation unfamiliarity or discomfort with, it also had the greatest increase in residents feeling comfortable or extremely comfortable (+48%). 

The five topics with the greatest decrease in percentage of residents who reported no familiarity or feeling not comfortable were all moderate to complex airway maneuvers and topics: flexible fiberoptic intubation (-72%), intubating LMA (-58%), laryngectomy (-49%), rigid bronchoscopy (-50%), and cricothyroidotomy (-48%) (p<0.001) (Figure 2). The mean decrease in percentage of residents reporting no familiarity or feeling not comfortable with these topics was 55%.

The percentage of residents who reported feeling unfamiliar or not comfortable with the ASA difficult airway algorithm prior to the boot camp was 70% (Figure 2). This decreased to 15% after completion of the boot camp (Figure 3). The percentage of residents who reported feeling comfortable or extremely comfortable with the ASA difficult airway algorithm prior to the boot camp was 3% (Figure 3). This increased to 50% after completion of the boot camp.

## Discussion

Simulation has been increasingly recognized as a useful tool to enhance the teaching of healthcare professionals as it represents a safe way to practice technical skills and poses no risks to patients. Simulation training is a tool that may be used to teach residents new skills and aid in their progression to competent independent physician. This transition is reflected in the Miller Model of Medical Competency in which expertise is built upon a pyramid of learning, with medical knowledge forming the pyramid’s base. Only upon gaining appropriate knowledge can trainees advance to the top of the pyramid where they are able to take action or perform advanced procedures based on clinical problems [7]. Simulation training also often incorporates ideas derived from the experiential learning cycle as described by Kolb which separates experiential learning into four separate processes: (1) thinking (abstract conceptualization), (2) applying (active experimentation), (3) experiencing (concrete experience), and (4) reflecting (reflecting observation) [8]. Accordingly, simulation has been proposed as being more effective when it replicates the work environment in a risk-free manner, has clear objectives and tools for learning, provides opportunity for deliberate practice, and allows for feedback and reflection after completion of the tasks.

Our data support the idea that an immersive boot camp experience can enhance resident familiarly and comfort with a wide range of airway tasks ranging from basic airway maneuvers to more complex procedures and topics. Our data show that the greatest improvement in comfort level was among the moderately complex airway topics.

As expected, the most complex airway topics and tasks also had close to or more than 90% of participants reporting no familiarity with them. These topics included laryngectomy, cricothyroidotomy, tracheostomy, and rigid bronchoscopy. All complex airway topics had decreases in the percentage of resident reporting no familiarity or feeling not comfortable with the topic after partaking in the simulation boot camp. These findings support that immersive simulation boot camp experiences are an excellent and effective method for introducing challenging clinical topics to novice trainees and decreasing novice trainee discomfort or lack of awareness with complex clinical problems. The value of awareness can be illustrated in the case of laryngectomy patients, where awareness by first responders of their unique airway can be lifesaving. 

Although junior otolaryngology and anesthesiology residents may be looked to as “experts,” the reality is that their experience level is that of a beginner or novice trainee. Many airway maneuvers of basic to moderate complexity can be lifesaving for patients, and our data support the early use of immersive boot camp training to decrease unfamiliarity and discomfort with airway management and enhance comfort levels. Every topic covered in our simulation boot camp, including the ASA difficult airway algorithm, resulted in statistically significant increases in comfort levels and decreases in discomfort and unfamiliarity upon completion of the boot camp experience. These findings echo those of other similar studies that evaluated the utility of boot camp training among novice surgical trainees [2,9-12].

A key limitation of our study is that our resident self-assessment tool is subjective in nature. Future studies should implement objective metrics by having residents demonstrate clinical skills or decision making with scoring by expert observers. Accordingly, further work is needed to examine whether the increased comfort level directly correlates with enhanced surgical skill or improvement in the time to competency. Another important limitation of our study is due to the anonymous nature of the self-assessment tool we were unable to distinguish between anesthesiology and otolaryngology resident scores. Future studies that are coded and tracked throughout a resident’s training could be used to pool data that may help identify trends to optimize customized and varied educational models for the residents. Nevertheless, the collective data provide support for the benefits of early exposure to immersive boot camp simulation training to help improve familiarity and comfort levels of residents with a topic as important and complex as airway management.

## Conclusions

Our data support the use of immersive surgical boot camp experiences to enhance resident familiarity and comfort and decrease unfamiliarity and discomfort with a wide variety of airway topics and maneuvers. The highest improvement in levels of comfortability was among the moderately advanced airway skills and concepts related to the ASA difficult airway algorithm. All airway topics covered in the course resulted in statistically significant improvement in percentage of participants’ comfort levels and statistically significant decreases in percentage of participants who reported no familiarity or discomfort with the topic. The greatest decrease in levels of discomfort was among the most complex airway situations and topics. 
